# CXCR6-CXCL16 axis promotes prostate cancer by mediating cytoskeleton rearrangement via Ezrin activation and α_v_β_3_ integrin clustering

**DOI:** 10.18632/oncotarget.6944

**Published:** 2016-01-19

**Authors:** Rajesh Singh, Neeraj Kapur, Hina Mir, Nalinaksha Singh, James W. Lillard, Shailesh Singh

**Affiliations:** ^1^ Department of Microbiology, Biochemistry & Immunology, Morehouse School of Medicine, Atlanta, GA, USA

**Keywords:** prostate cancer, CXCR6, CXCL16, Ezrin, α_v_β_3_ integrin

## Abstract

Cytoskeletal rearrangement is required for migration and invasion, which are the key steps of cancer metastasis. Ezrin and integrin co-ordinate these processes by regulating cellular adhesion and cytoskeletal polymerization-depolymerization. It is also well established that chemokine-chemokine receptor axis plays a crucial role in regulating cancer cell migration and invasion. In this study, we show involvement of CXC chemokine receptor 6 (CXCR6) and its only natural ligand CXCL16 in pathobiology of prostate cancer (PCa). CXCR6 is highly expressed in PCa tissues and cell lines (LNCaP and PC3), relative to normal tissue and cells. CXCR6 expression in PCa tissues correlated with higher Gleason score. Similarly, aggressive PCa cells (PC3) show high CXCR6 compared to less aggressive LNCaP. Besides, PC3 cells show higher MMPs expression compared to LNCaP cells following CXCL16 stimulation. Intriguingly, CXCR6-CXCL16 interaction in PCa cells promotes Ezrin activation, &alpha;_v_&beta;_3_ integrin clustering and capping at the leading edge in FAK/PI3K/PKC dependent manner, thereby modifying cellular adhesion as well as motility. Together these results demonstrate that CXCL16 stimulation changes cytoskeletal dynamics resulting in enhanced migration, invasion and adhesion to endothelial cells, ultimately enabling PCa cells to achieve their metastatic goal.

## INTRODUCTION

Tumor cells migrate to distant sites in response to chemokines via interaction with corresponding chemokine receptors [1–5]. Many different chemokine receptors such as CXCR4, CXCR5, CCR2, CCR5, CCR9 and others have been shown to be involved in progression and metastasis of different solid and hematologic malignancies including prostate cancer (PCa). Recently, it has been shown that CXCR6 and its only natural ligand CXCL16 are over-expressed in PCa [6, 7], and are responsible for the enhanced proliferation and invasion of PCa cells [8]. PCa is an indolent disease characterized by change in the morphology and adhesion properties of cells in transition from normal to prostatic intraepithelial neoplasia (PIN) and eventually invasive cancer. Although activation of MMPs underscore the invasive capability of metastatic cell, cytoskeletal rearrangement is pre requisite for a cell to detach itself from primary site and adhere on specific cell matrices [9]. Actin rearrangement plays a key role in regulating cellular processes such as motility and vesicular trafficking. Actin filament-binding proteins further regulate cell migration [10]. Ezrin is a linker protein involved in PCa pathobiology and supports linking of cell membrane proteins with actin cytoskeleton [11–13]. Apart from cellular migration, this interaction provides an intracellular scaffold for the formation of specialized membrane domains that facilitate signal transduction through a number of growth factor receptor, including androgen receptor, and adhesion molecules that are important for tumor development and progression [11]. Cell adhesion to the extracellular matrix (ECM) involves the coordinated assembly and disassembly of integrins into complexes called focal adhesions. In these complexes, the internal tails of integrin β-subunits are typically linked to the actin cytoskeleton via cytoplasmic proteins, involved in scaffolding, adaptor, regulatory and mechano-transduction functions [14, 15]. In the present study, we have provided the evidence that CXCR6-CXCL16 interaction mediates metastatic process in PCa via regulating Ezrin-Actin polymerization and integrin clustering.

## RESULTS

### Expression of CXCR6 in PCa tissues and cell lines

**Figure 1:**
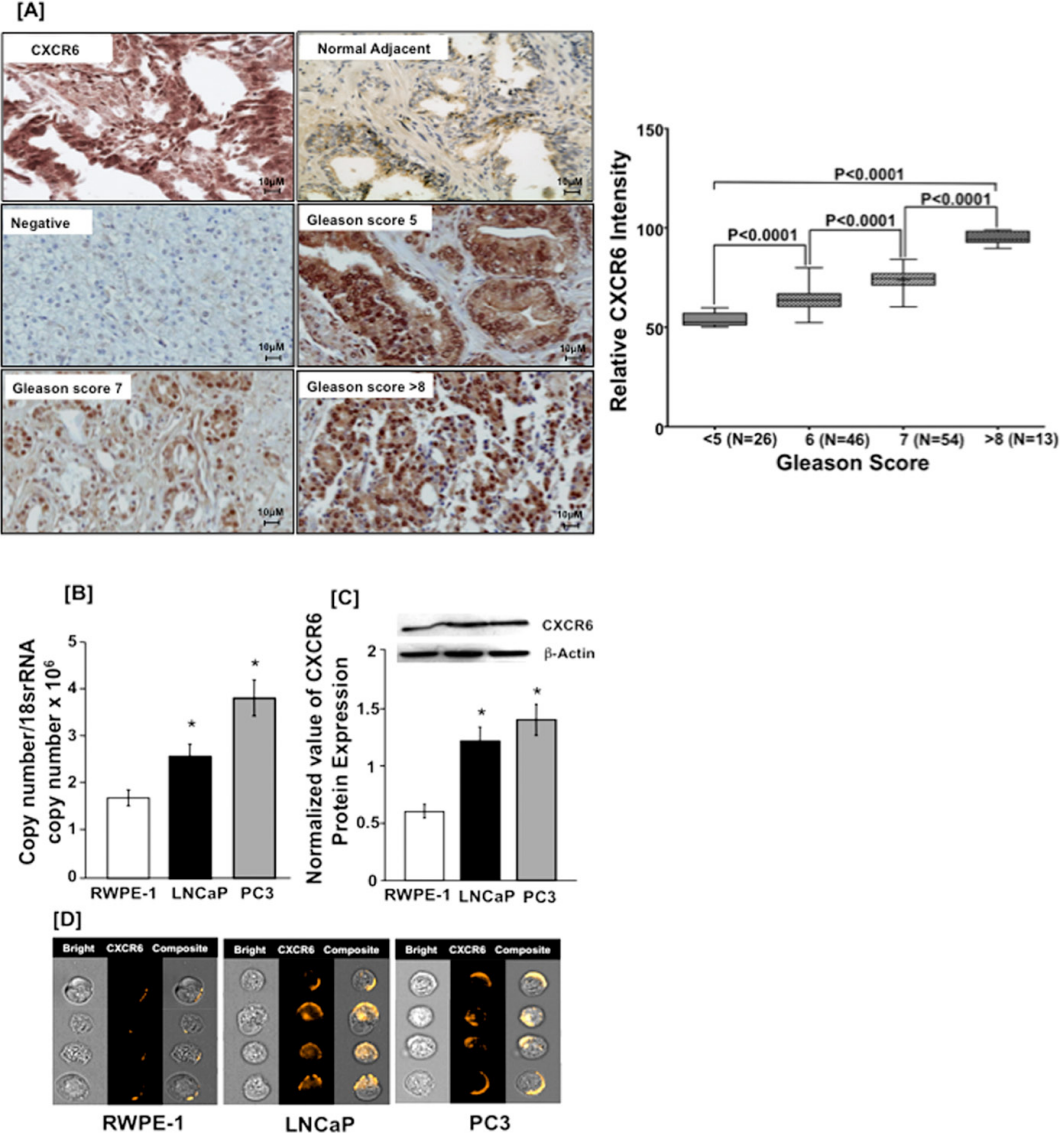
CXCR6 expression in prostate cancer tissue and cell lines **Panel A.** PCa tissue microarray was stained for CXCR6. Brown color represents DAB staining of CXCR6 and blue color represents nuclei stained with Hematoxylin. Image labeled ‘Negative’ represents the non-specific binding of secondary antibody. The differences in relative CXCR6 expression of prostate tumor with respect to Gleason Score (≤5, 6, 7 and ≥ 8) were analyzed using Mann Whitney U test and were significantly different (p < 0.0001). Each box in the plot represents minimum and maximum CXCR6 immunointensity and line in the box indicate the median CXCR6 immunointensity for the respective Gleason group. **Panel B.** Quantitative RT-PCR analysis of CXCR6 mRNA expression was performed in triplicate in PCa cell lines- PC3 (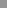) and LNCaP (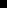) as well as from RWPE-1 cells (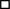). Copies of transcripts are expressed relative to actual copies of 18S rRNA ± SE. **Panel C.** Western blot was performed three times using total cellular protein isolated from PC3 (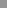), LNCaP (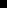) and RWPE-1 cells (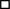). Ratio of integrated density of CXCR6 and β-Actin of respective sample were taken and results are expressed as ratio ± SE. **Panel D.** PCa and RWPE-1 cells were stained with PE conjugated anti-human CXCR6. Cells were imaged by Image stream. Asterisk (^*^) indicates statistical significance (*p* < 0.05) between normal and cancer cells.

Emerging evidence suggests that CXCR6 expression is responsible for cancer progression [16]. Using tissue microarrays we found that expression of CXCR6 was higher in PCa tissue than normal adjacent. PCa tissues displayed membrane, cytoplasmic as well as nuclear localization of CXCR6 whereas normal adjacent tissue showed weak cytoplasmic staining. Expression of CXCR6 in PCa tissue correlated with Gleason score (Figure 1A). PCa tissues with Gleason scores >8 displayed predominantly nuclear and to a lesser degree membrane and cytoplasmic CXCR6 expression patterns. PCa cases with Gleason score 5 for the most part displayed membrane and cytoplasmic, but minimal nuclear CXCR6 expression patterns. Expression of CXCR6 in PCa cell lines (LNCaP and PC3) and normal prostatic epithelial (RWPE-1) cells were determined at mRNA and protein level using qRT-PCR and, western blot and immunofluorescence, respectively. CXCR6 transcripts in both PCa cell lines were significantly higher than RWPE-1. Further, CXCR6 mRNA expression in PC3 was found to be higher than in LNCaP cells (Figure 1B). Similarly, western blot showed significantly low level of CXCR6 protein in RWPE-1 as compared to PC3 and LNCaP cells (Figure 1C). These results were further confirmed by Amnis Image stream, an image based flow cytometer system. Images obtained after spectral correction showed less expression of CXCR6 in RWPE-1 cells compared to PC3 and LNCaP cells (Figure 1D). Therefore, these findings suggest association of CXCR6 with PCa. Further experiments were done to understand the significance of this association.

### CXCR6-CXCL16 interaction promotes PCa cell migration and invasion

**Figure 2:**
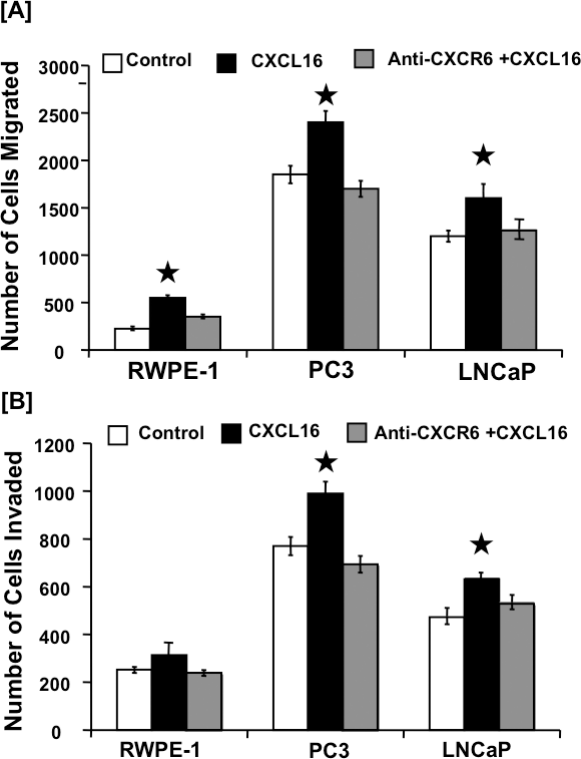
CXCR6-mediated cell migration and invasion **Panel-A.** CXCR6-mediated PCa cell migration. PC3, LNCaP, and RWPE-1 cells were tested for their ability to migrate towards chemotactic gradients of 100 ng/ml CXCL16 (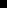), or 100 ng/ml CXCL16 after blocking CXCR6 with 1 μg/ml anti-CXCR6 antibody (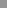) and compared to their migration in the absence of CXCL16 as a chemo attractant (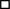). Asterisk (^*^) indicates significant differences (*p* < 0.05) between no additions and CXCL16-treated cells. **Panel-B.** CXCR6-mediated PCa cell invasion. PCa and RWPE-1 cells were tested for their ability to invade or translocate across a Matrigel matrix in response to chemotactic gradients with (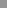) or without (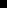) blocking CXCR6 using 1 μg/ml anti-CXCR6 antibody and no CXCL16 as chemo attractant used as control (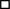). Asterisk (^*^) indicates significant differences (*p* < 0.05) between no additions and CXCL16-treated cells.

The functional significance of CXCR6-CXCL16 axis in PCa was evaluated using migration and invasion assays. Higher number of PC3 and LNCaP cells migrated, and invaded through matrigel under chemotactic gradient of CXCL16, which was significantly inhibited by blocking CXCR6-CXCL16 interaction with anti-CXCR6 antibodies (Figure 2A). PC3 cells, which have higher CXCR6 expression, showed higher migratory and invasive potential than LNCaP cells. We observed very less number of normal prostatic epithelial cells (RWPE-1) migrating and invading in response to CXCL16 gradient and these cells showed weak CXCR6 expression (Figure 2B). These findings suggest that CXCR6-CXC16 axis is functional in PCa cells and could promote their exodus to distant sites.

### CXCR6-CXCL16 induced active MMP expression in PCa cell lines

**Figure 3:**
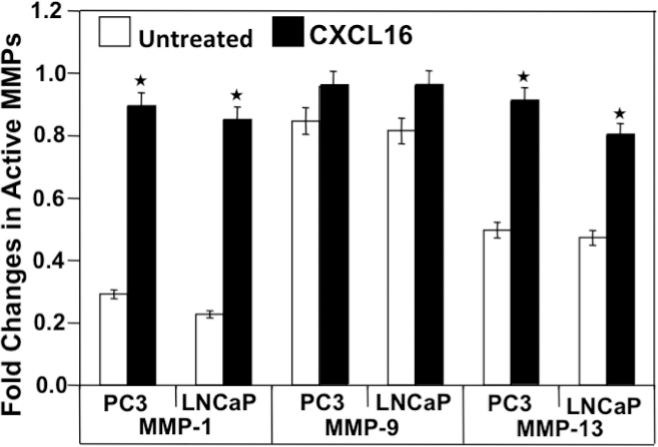
CXCL16 induced active MMP expression by PC3 and LNCaP cells Cells were cultured for 24 h with (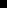) or without (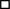) CXCL16 (100 ng/ml). Total and active MMP-1, -9, and -13 protein levels were determined by ELISA. Asterisk (^*^) denotes a significant change in active/total MMPs level (p < 0.05) induced by CXCR6-CXCL16.

MMPs are a large family of proteolytic enzymes that degrade the extracellular matrix and basement membrane and hence, play an important role in cancer invasion and metastasis [17]. To determine if the associated increase in invasion after CXCL16 induction is due to heightened MMP activity, levels of active collagenase (MMP-1 and -13) and gelatinase (MMP-9) were determined by ELISA. A significant increase in the active MMP-1 and -13 expressions was observed in CXCL16-treated LNCaP and PC3 cells in comparison to untreated cells. Increase in active MMP-9 following CXCL16 stimulation in both PCa cell lines was not highly significant (Figure 3). Taken together these results suggest that CXCR6-CXCL16 axis promotes cell invasion by modulating MMPs expression.

### CXCR6 stimulation promotes cell migration by Ezrin phosphorylation in PCa cell lines

**Figure 4:**
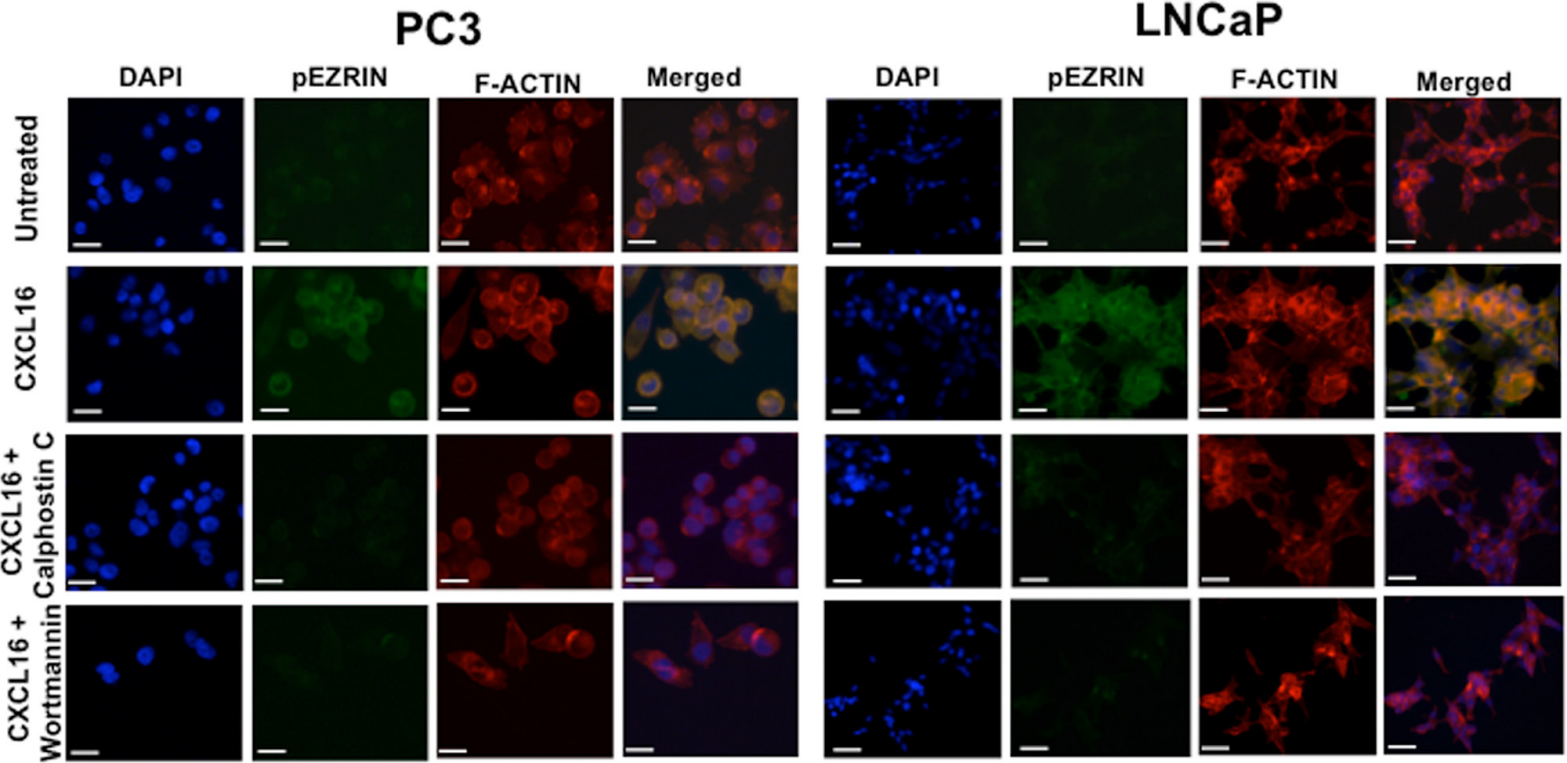
CXCR6-CXCL16 dependent Ezrin phosphorylation in PCa cells PC3 and LNCaP cells cultured on cover slips were treated with 100ng/ml CXCL16 for 5 min with or without Calphostin C (100 nM) or Wortmannin (10 μM) pretreatment. Cells were stained with Rhodamine Phallodin and Alexa Fluor 488 conjugated Mouse anti-Ezrin (pY353) antibody. Images were captured using Olympus FluoView FV1000 confocal microscope with 60X oil immersion objective. Scale bar represents 20μm.

We subsequently determined if CXCR6-mediated migration and invasion is via activation of Ezrin, which is known to regulate invadopodia formation. PC3 and LNCaP cells, after treatment with CXCL16, showed an increase in p-Ezrin and expression of F-Actin (Figure 4). Increase in p-Ezrin and F-Actin in PCa cells was inhibited when cells were pre-treated with 100nM PKC inhibitor (Calphostin C) or 10μM PI3K inhibitor (Wortmannin) 2 hours prior to CXCL16 stimulation (Figure 4). This shows that CXCR6-CXCL16 interaction rearranges the cytoskeletal proteins to enhance PCa cell migration and invasion by activating PKC and PI3K pathway, although in-depth studies will be needed to define the molecular details. An interesting observation was that phosphorylation of Ezrin was higher in LNCaP cells compared to PC3 cells; significance of this is discussed later.

### CXCR6-CXCL16 axis induces α_v_β_3_ integrin clustering in PCa cells

**Figure 5:**
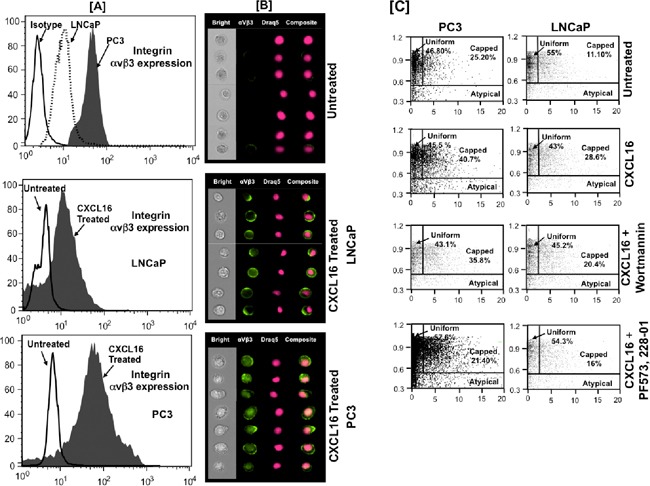
CXCR6-CXCL16 induced α_v_β_3_ integrin clustering in PCa cells **Panel-A.** Expression of integrin α_v_β_3_ in PCa cell lines. Untreated or CXCL16 treated PCa cells stained for integrin α_v_β_3_ and nucleus stained with Draq5. **Panel-B.** Quantitative analysis of CXCL16 induced integrin α_v_β_3_ clustering using ImageStream100 image based flow cytometer. Bright field (white), Integrin α_v_β_3_ (green) and Draq5 (red), composite images for representative PC3 and LNCaP cells are shown. **Panel-C.** Percentage of cells showing α_v_β_3_ integrin clustering in response to CXCL16 treatment with or without PKC/FAK inhibition. Integrin clustering was quantified using two standard image-based software analysis features: Area aspect ratio and Radial delta centroid, which demonstrated three types of cell population in samples: Uniform (global distribution of integrin α_v_β_3_ on cell surface; cells with high aspect ratio with low radial delta centroid values), Capped (integrin α_v_β_3_ clustered in a specific cell area; cells with high aspect ratio values and relatively low radial delta centroid) and Atypical (cell debris; cells with smaller aspect ratio and low radial delta centroid).

We analyzed the effect of CXCL16 on α_v_β_3_ integrin clustering and change in cellular morphology. We observed increased α_v_β_3_ integrin expression in CXCL16 treated LNCaP and PC3 cells compared to untreated cells. Further, PC3 cells showed increased α_v_β_3_ integrin expression compared to LNCaP cells after CXCL16 treatment, which could be because of its higher basal expression in these cells (Figure 5A & 5B).

As shown in Figure 5C, we further characterized the cell population by plotting the Radial Delta Centroid against the Bright field Aspect Ratio feature of Amnis Image Stream. The visual inspection of bright field imagery and fluorochrome distribution patterns allowed us to define morphologically distinct types of cells. Cells in which α_v_β_3_ integrin was uniformly distributed on the surface were labeled as ‘uniform’ and the cell population was defined as ‘capped’ where α_v_β_3_ integrin was clustered at one loci of cell surface. In addition to this we also noted population of cells without proper integrin distribution. This distinct morphological group of cells was grouped as “Atypical”. Visual inspection of these revealed them to be cellular debris. Our data shows that percentage of capped cells was higher in PC3 cells than in LNCaP cells and CXCL16 treatment increases this by ~1.5-2.5 fold. This induction of capping was prevented when cells were treated with PI3K (Wortmannin) and FAK (PF573228) inhibitors. With FAK inhibition, CXCR6-CXCL16 induced capping was reduced to ~50% in both PC3 and LNCaP. However, reduction in capping after PI3K inhibition in PC3 and LNCaP was ~10% and −30%, respectively. These data imply that CXCR6-CXCL16 not only reorganize the actin filaments but also promotes α_v_β_3_ integrin clustering via PI3K and FAK pathway. Consequently, CXCR6-CXCL16 mediated morphological changes, which were higher in more aggressive PC3 compared to LNCaP, could be due to differential activation of FAK and PI3K in PC3 and LNCaP.

### CXCR6-CXCL16 dependent adhesion of PCa cells to HBME cells

**Figure 6:**
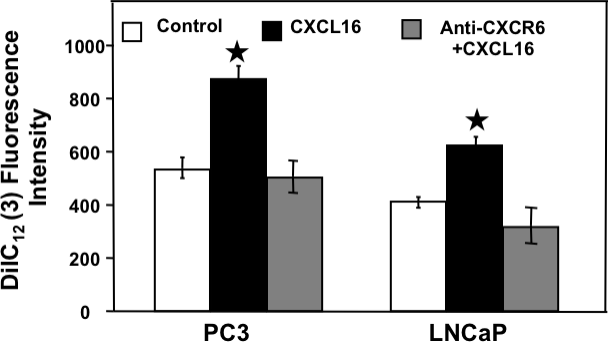
CXCR6-CXCL16 dependent adhesion of PCa cells with Human Bone marrow endothelial cells Untreated (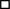), CXCL16 treated with (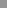) or without CXCR6 blockade (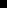) were incubated with DilC_12_ labeled human bone marrow endothelial cells (HBME) cells for 30 min. Attached cells were counted by measuring fluorescence of DilC_12_ (3) at 549/565nm (Ex/Em) after washing off the unattached HBME cells. Results are expressed as mean ± SE. Asterisk (^*^) indicates significant difference (p < 0.05) in PCa cell adhesion between treated and control cells.

Having determined that CXCR6-CXCL16 interaction is responsible for the integrin α_v_β_3_ clustering, we investigated the role of CXCR6-CXCL16 axis in adhesion of LNCaP and PC3 cells to HBME cells, a model often used to study the role of integrin clustering on bone metastasis of PCa cells [18]. Treatment of PCa cells with CXCL16 significantly enhanced their attachment with HBME cells. This adhesion was significantly reduced by CXCR6 blockade (Figure 6), which suggests involvement of CXCR6-CXCL16 axis in PCa cell adhesion and explains their preferential metastasis to bone.

## DISCUSSION

Chemokines/chemokine receptors serve as guiding sticks for cancer cell metastasis [4, 5, 19–22]. Although CXCR4 has been well studied, CXCR6 has also emerged as a major chemokine of interest in cancer [21]. Despite numerous studies associating CXCR6 and CXCL16 with PCa [6, 7], a complete picture of how this interaction boosts the disease progression is yet to be defined. Here we show higher expression of CXCR6 in PCa tissues and cell lines, relative to their respective controls. These results corroborate with earlier findings and suggest that CXCR6 expression is associated with tumorigenic transformation in PCa. In this study, we explored the molecular mechanisms contributing to major steps involved in metastasis.

Cell migration through extracellular matrix (ECM) is necessary for cancer cells to invade adjacent tissues and metastasize to distant organs. Since CXCR6-CXCL16 interaction have been linked with invasion of cancer cells [21, 23, 24], we evaluated CXCR6-CXCL16 dependent mechanisms instigating migration and invasion of PCa cells. Migration and invasion of PCa cells induced by CXCL16 could be abrogated by anti-CXCR6 antibody. Increased expression and activity of MMPs, particularly the collagenases, execute this effect of CXCL16. However, to determine if the observed increase in MMP activity is a consequence of change at an upper hierarchy, we scrutinized the effect of CXCL16 on cytoskeleton rearrangement. In this regard, we focused on Ezrin, a protein that can bind F-actin, membrane proteins and other signaling molecules, and regulate cytoskeleton rearrangement when phosphorylated [25]. Interaction of Ezrin with cytoskeleton also provides an intracellular scaffold for the formation of specialized membrane domains that facilitate signal transduction through a number of adhesion molecules thus regulating cell survival, adhesion and migration/invasion [12]. Ezrin is up regulated in PCa tissues and cells [13, 18, 26, 27]. Sequential phosphorylation of Ezrin in PCa cells, at Thr 566 and Tyr 353, occurs in androgen dependent manner through the activation of protein kinase C (PKC) and Src [12]. This could be the reason behind higher Ezrin phosphorylation observed in androgen responsive LNCaP cells than androgen insensitive PC3 cells. In other words, phosphorylation of Ezrin in LNCaP cells could be a cumulative effect of CXCL16 and androgen. At the same time, based on Ezrin phosphorylation observed in response to CXCL16 in PC3, it could be inferred that CXCR6-CXCL16 interaction may cause cytoskeleton rearrangement and promote tumor cell metastasis independent of androgen.

Phosphorylated Ezrin coalesces into a cap on the cell surface, defining the location for pseudopod formation. Cell then elongates, creating a familiar proboscis-like structure and ‘Podo’ is almost entirely localized in the new pseudopod. Ezrin functions downstream in this signaling initiated at transmembrane receptor-integrins [28]. Integrins connect ECM to cytoskeleton allowing the cell to endure the pulling force to increase the integrin–ligand association by inducing a high-affinity conformational change [29]. Clustering of integrin leads to cytoskeletal protein rearrangement and formation of focal adhesion complexes that eventually promote cell motility. Numerous studies has documented that disseminated PCa cells are characterized by altered integrin expression [30–32]. In particular, α_v_β_3_ integrin, which is not expressed in normal prostate tissue but is up regulated in prostatic adenocarcinoma, has been linked to their invasive behavior [33]. It has also been reported that α_v_β_3_ integrins are important in controlling growth and metastasis of PCa cells to bone. Over expression of α_V_ subunit enhances PCa cell adhesion to type I collagen, fibronectin and laminin, proteins that are commonly found in bone microenvironment [32]. In our study, increased phosphorylation of integrin -β_3_, activation of integrin pathway (α_v_β_3_ pathway of integrin signaling) and increased α_v_β_3_ clustering in PCa cells following CXCL16 treatment; and enhanced adhesion of PCa cells to HBME cells in presence of CXCL16 emphasizes that CXCR6-CXCL16 axis plays a crucial role in trafficking and homing of PCa cells. Studies has shown that CXCL12 also induces α_v_β_3_ integrin clustering [34, 35], however clustering observed in our study is CXCL16 specific as CXCR6 blockade decreases PCa cell adhesion to HBME cells. It further shows significance of this axis for maintenance of aggressive phenotype and also signifies of studying chemokines other than CXCR4 in cancer progression to define the disease process and develop better therapeutic interventions.

Studies on melanoma, glioma and angiogenic endothelial cells show that integrin α_v_β_3_ associates with MMP-activating multi-protein complex to regulate/activate MMP [36] besides regulating cellular adhesion. Similar role of α_v_β_3_ integrin in regulating MMPs has been shown in chondrosarcoma cells [37]. Hence the observed increase in MMP-13 activity in our study might be associated with increased clustering of α_v_β_3_ following CXCL16 stimulation. It is also clear from our data that CXCL16 induces its effect on capping and migration via FAK activation, which in turn could affect PI3K dependent and independent pathways. PKC could also play role in this CXCL16 mediated effect independent of or in conjunction with PI3K. Reports also show that integrin and Ezrin operate through PI3K/Akt pathway. In all, our results imply that CXCR6 supports PCa development via FAK/PI3K/Akt pathways.

In conclusion, this study provides strong evidence that CXCR6-CXCL16 interaction is involved in Ezrin activation and integrin clustering that changes the morphology and adhesion properties of PCa cells. These cellular rearrangements regulated by CXCL16, altogether promote invasion and migration of PCa cells by activating FAK/PI3K/Akt pathways. Hence, higher CXCR6 expression in PC3 cells and consequent higher capping due to increased α_v_β_3_ integrin expression than in LNCaP cells suggests that differential CXCR6 expression could correlate with differential molecular rearrangements required for PCa cell to disseminate, travel and home at the distant sites.

## MATERIALS AND METHODS

### Immunohistochemistry

PCa tissue micro-arrays (TMA) along with associated clinical pathology [i.e., PCa (*n* = 139) and Normal Adjacent (*n* = 12) cases] were obtained from the National Cancer Institute (NCI) Cooperative PCa Tissue Resources (CPCTR). Specifically, PCa cases (*n* = 139) consisted of samples having Gleason scores of ≤ 5 (*n* = 26), 6 (*n* = 46), 7 (*n* = 54), >8 (*n* = 13). Normal adjacent tissues were not available for all of the PCa cases used for analysis. TMA was stained for CXCR6. Briefly, TMA was de-paraffinized, rehydrated and incubated with 0.3% H_2_O_2_ to block the endogenous peroxidase activity. Following washing with de-ionized water and Tris-buffer (pH 7.6) the slide was incubated with Fc block (Innovex Biosciences, CA, USA) for 30 minutes at room temperature (RT) in a humidity chamber. Sections were washed with Tris buffer and incubated with 3% normal goat serum for 1 h at RT to reduce non-specific binding. Next, sections were incubated with anti-CXCR6 antibody (R&D Systems, USA) at 1:50 dilution for 1 h at RT. After washing, sections were incubated with multispecies link at RT for 20 minutes, washed and developed with a 3,3′-diaminobenzidine (DAB, Biogenex, USA). Sections were counterstained with Mayer's hematoxlin (Sigma) for 1 minute, then de-hydrated and mounted with permount (Sigma). Digital images were captured and analyzed using an Aperio ScanScope scanning system (Aperio Technologies, USA).

### Cell lines and cell culture

Human prostate cancer cell lines (PC3 and LNCaP) and normal prostatic epithelial cell line (RWPE-1) were obtained from American Type Cell Culture (ATCC). Human bone marrow endothelial (HBME) cells were kind gift from Dr. Kenneth Pienta, (University of Michigan, MI, USA). PC3 cells were cultured in F-12K medium with 2 mM L-glutamine and 10% fetal bovine serum (FBS). LNCaP cells were cultured in RPMI supplemented with 10% FBS. RWPE-1 cells were cultured in Keratinocyte Serum Free Medium kit (Invitrogen, USA). HBME cells were cultured in DMEM supplemented with 100 μg/ml of streptomycin, and 100 U/ml of penicillin and 10% FBS. All cell lines were cultured at 37°C with 5% CO_2_.

### RNA isolation and gene expression analysis

Total RNA was isolated using Trizol method and quantitative RT-PCR was performed to evaluate the expression of CXCR6 and 18S genes as described earlier [4, 5]. Copy number of CXCR6 mRNA was calculated following normalization with 18S rRNA expression [38].

### Western blotting

Expression of CXCR6 protein in LNCaP, PC3 and RWPE-1 cells was confirmed by western blotting following the protocol described previously [19]. Briefly, equal amount of protein (50 μg/lane) was resolved by electrophoresis and transferred to PVDF membrane (BioRad). Following blocking, the membrane was incubated overnight at 4°C with anti-CXCR6 primary antibody (Ray Biotech) at 1:500 dilution. Subsequently, the membrane was washed and incubated with HRP conjugated anti-rabbit IgG (R&D systems) at 1:2000 dilution for 1 h at RT. Following incubation, the immune-reactive bands were detected on Hyperfilm-ECL by ECL Plus reagent (GE Healthcare Bio-Sciences). After detection of CXCR6, the membrane was stripped using stripping buffer (Pierce, USA) for 15 min at RT; washed, blocked and probed for β-actin. ImageJ software (http://www.rsbweb.nih.gov/ij) was used to quantify the results from two independent experiments.

### Migration and invasion assays

Migratory and invasive potential of PC3, LNCaP and RWPE-1 cells were evaluated using BD BioCoat migration or matrigel invasion chambers (BD Labware, NJ, USA) respectively, as described in previous studies [20, 22]. Briefly, 2×10^4^ cells were added to the top chamber of inserts and allowed to migrate under chemotactic gradient of recombinant human CXCL16 (100 ng/mL; PeproTech, NJ, USA) added to the bottom chamber as chemo-attractant. To verify that observed responses are CXCR6 dependent, cells were pre-incubated with 1.0 μg/mL mouse anti-human CXCR6 antibody (R&D Systems) for 1 h before the assay. The cells, with and without anti-CXCR6 antibody, were incubated for 16 h at 37°C in 5% CO_2_. Following incubation, cells were fixed with 100% methanol and stained with 1% toluidine blue supplemented with 1% borax. Stained cells were counted by microscope at 40X magnification.

### Total and active MMP ELISA

Cells were seeded in 24-well plates and cultured for 24 h with or without CXCL16 (100 ng/mL). Total and active MMP-1, MMP-9 and MMP-13 were quantified in conditioned media using Flurokine ELISA Kit (R&D Systems), according to the manufacturer's protocol. Changes in the active MMP level was calculated by dividing the active MMP with total MMP level.

### Microscopy

PC3 and LNCaP cells were cultured on Poly L-Lysine coated coverslips in RPMI with 10% FBS, which was replaced 24 h before the experiment with RPMI containing 2% FBS. For the PI3K/FAK inhibition, cells were incubated with either Calphostin C (100 nM) or Wortmannin (10 μM) 2 h before addition of CXCL16. The cells were fixed with 4% paraformaldehyde in PBS for 10 minutes after 5 minutes CXCL16 treatment. After fixation cells were permeabilized for 5 minutes with permeabilization buffer (Cytoskeleton Inc. Denver). After washing with wash buffer (Cytoskeleton Inc. Denver), cells were incubated for 40 minutes with 100nM Rhodamine Phallodin and 20μl Alexa Fluor 488 conjugated Mouse anti-Ezrin (pY353) (BD Biosciences). After three washings the coverslip was mounted on slide in presence of aqueous permanent mounting medium (DAKO). Images were captured using Olympus microscope with 60X oil immersion objective.

### Image based flow cytometry analysis

#### CXCR6 expression

Expression of CXCR6 in PC3, LNCaP and RWPE-1 were analyzed by image based flow cytometry. Cells were incubated with 1 μg of Fc Block (PharMingen, CA, USA) per 10^6^ cells for 15 min at RT and then stained with 10 μl (25 mg/ml) of PE-conjugated mouse anti-human CXCR6 (R&D system) or mouse IgG2a isotype control antibody (R&D system) at 4°C for 40 min. The images were acquired using Image stream system and analyzed after spectral correction using Image Data Exploration and Analysis Software (IDEAS) (Amnis, USA).

### CXCR6-CXCL16 dependent α_v_β_3_ integrin clustering and cell morphology

PCa cells were treated with 100 ng/ml recombinant hCXCL16 (Peprotech) for 5 min. Staining was done as described above. Briefly, the cells were stained with 1 μg of FITC-conjugated mouse anti-human α_v_β_3_ antibody (BD PharMingen) at 4°C for 30 min. Nuclei of the cells were stained with 0.5 μl/ml Draq5 (Axxora, CA, USA). After staining, cells were fixed with 2% PFA for 10 min at 4°C. PCa cells stained using anti-human α_v_β_3_ antibody were imaged by Image stream system (Amnis, WA, USA) and analyzed using Image Data Exploration Analysis (IDEA) software. This system can also distinguish between rounded and elongated cells based on calculation of Aspect Ratio value (AR). The AR value in the bright field image is defined as the ratio of minor axis (width) to major axis (height) and is 1.0 for circular objects, while elongated cells have values significantly less than one. This was calculated using the Radial Delta Centroid (RDC) feature of IDEA (Image Data Exploration and Analysis) software. RDC is estimated by measuring the radial distance between the geometrical centers of the nuclear image and the fluorescent image stain. The RDC is then calculated using the Pythagorean theorem. DNA was stained with 1,5-Dihydroxyanthraquinone (DRAQ5; fluorescent DNA binding dye) and Podo with fluorochrome conjugated antibodies [37]. If the Podo protein is uniformly distributed throughout the membrane and is visible around the nucleus, the RDC value is smaller than the case when the ‘Podo’ protein is capped to one side of the membrane. Plotting the RDC between the nuclear and ‘Podo’ image distribution together with the aspect ratio of the bright field image allows identification of three cellular populations: i) capped cells (‘podo’ staining at one pole of the cell), ii) rounded population (non-elongated with ‘Podo’ staining uniformly distributed in membrane) and iii) Atypical cells [37]. Uniformly labeled cells had high Aspect Ratio but low Radial Delta Centroid values. Capped cells had high Aspect Ratio values but relatively low Radial Delta Centroid values. Using these classifiers, the percentage of each population was quantified. Three different cell populations were confirmed by representative image galleries [37]. The results were analyzed by use of Radial Delta Centroid (RDC) feature of IDEAS® software (Amnis) after spectral correction by Inspire® software (Amnis).

### Cell adhesion assay

HBME cells (5 × 10^4^ cell/well) were seeded in 24 well plates and allowed to grow up to 80% confluency. Next day, the cells were incubated with 10 mg/ml DilC_12_ (3) fluorescent dye solution (BD Biosciences, MA, USA) for 30 min in presence of 5% CO_2_ at 37°C. Cells were washed with serum free RPMI medium to remove unbound DilC_12_ (3). At the same time, LNCaP and PC3 cells (10^5^ cells in 500 mL) were treated with 100 ng/ml of CXCL16 or with 1.0 μg/mL of anti-CXCR6 Ab 1 h before the treatment with 100 ng/ml CXCL16. Untreated cells were used as control. Thereafter, PCa cells were added in the wells containing HBME cells and allowed to adhere for 30 min in presence of 5% CO_2_ at 37°C. Subsequently, non-adherent cells were removed by aspirating the media from wells and washing thrice with HBSS. Plates were read using a fluorescence reader at excitation and emission of 549 nm and 565nm, respectively.

### Statistics

Comparisons of CXCR6 immunointensity in Prostate TMA were made by non-parametric Mann Whitney U test. Results were declared significant at α level of <0.05. Expression of CXCR6, MMPs, mRNA and/or protein in PCa cell lines was compared using a two-tailed Student's t-test and expressed as means ± SEM. Results of adhesion, migration and invasion assays were analyzed by one-way ANOVA. Values were declared significantly different at a level of 0.05.

## Abbreviations

PCa: Prostate Cancer
MMP: Matrix metalloproteinase
PI3K: Phosphatidylinositol-4, 5-bisphosphate 3-kinase
